# Supplementary figures and images for: The Duck RXRA Gene Promotes Adipogenesis and Correlates with Feed Efficiency
Source: Animals (Basel). 2023 Feb 15;13(4):680. doi: 10.3390/ani13040680 (PMC9952354; doi:10.3390/ani13040680)

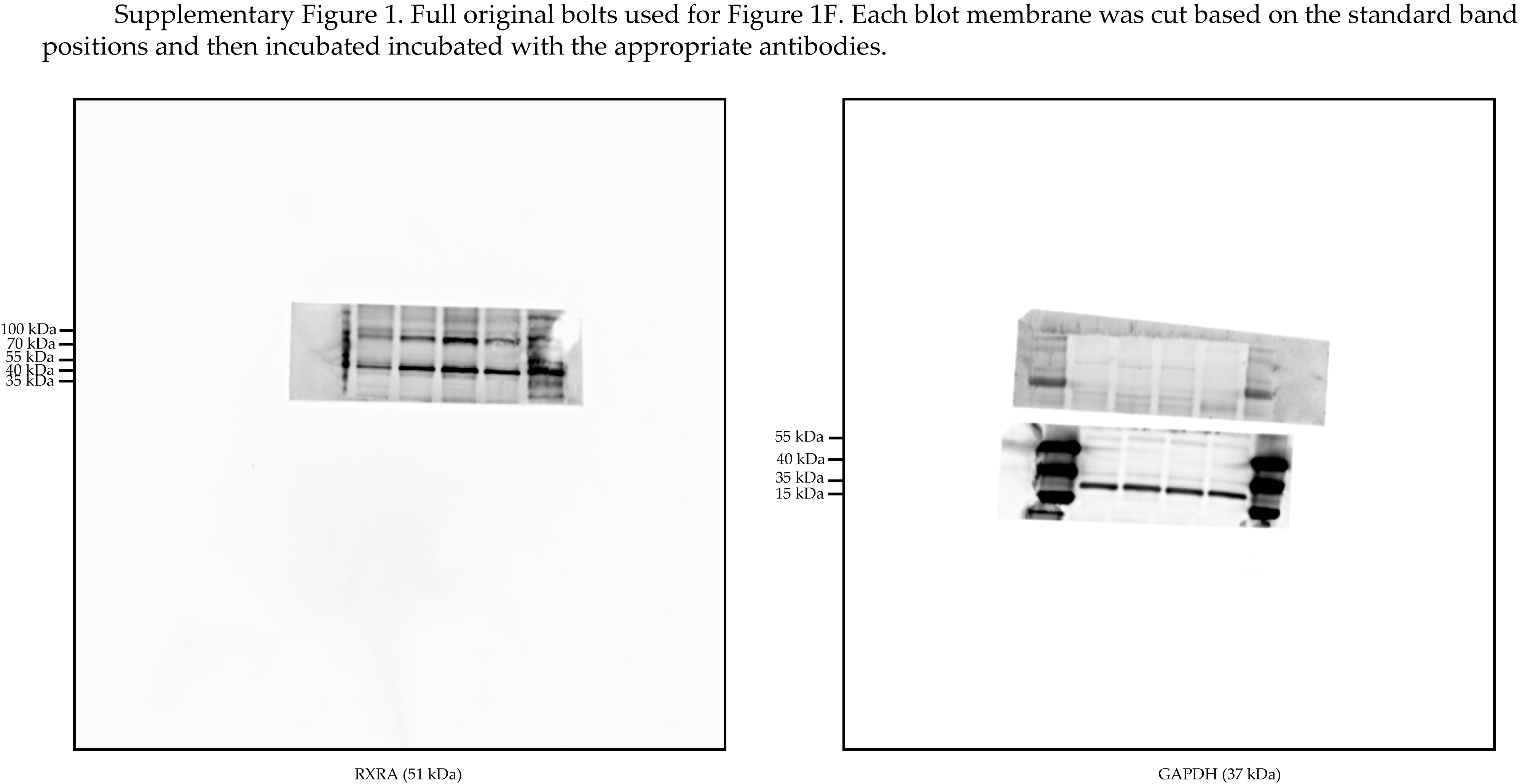

Supplement: Supplementary file 1 [file animals-13-00680-s001.zip › Supplementary materials/Figure S1-Full original bolts used for Figure 1F.tif]

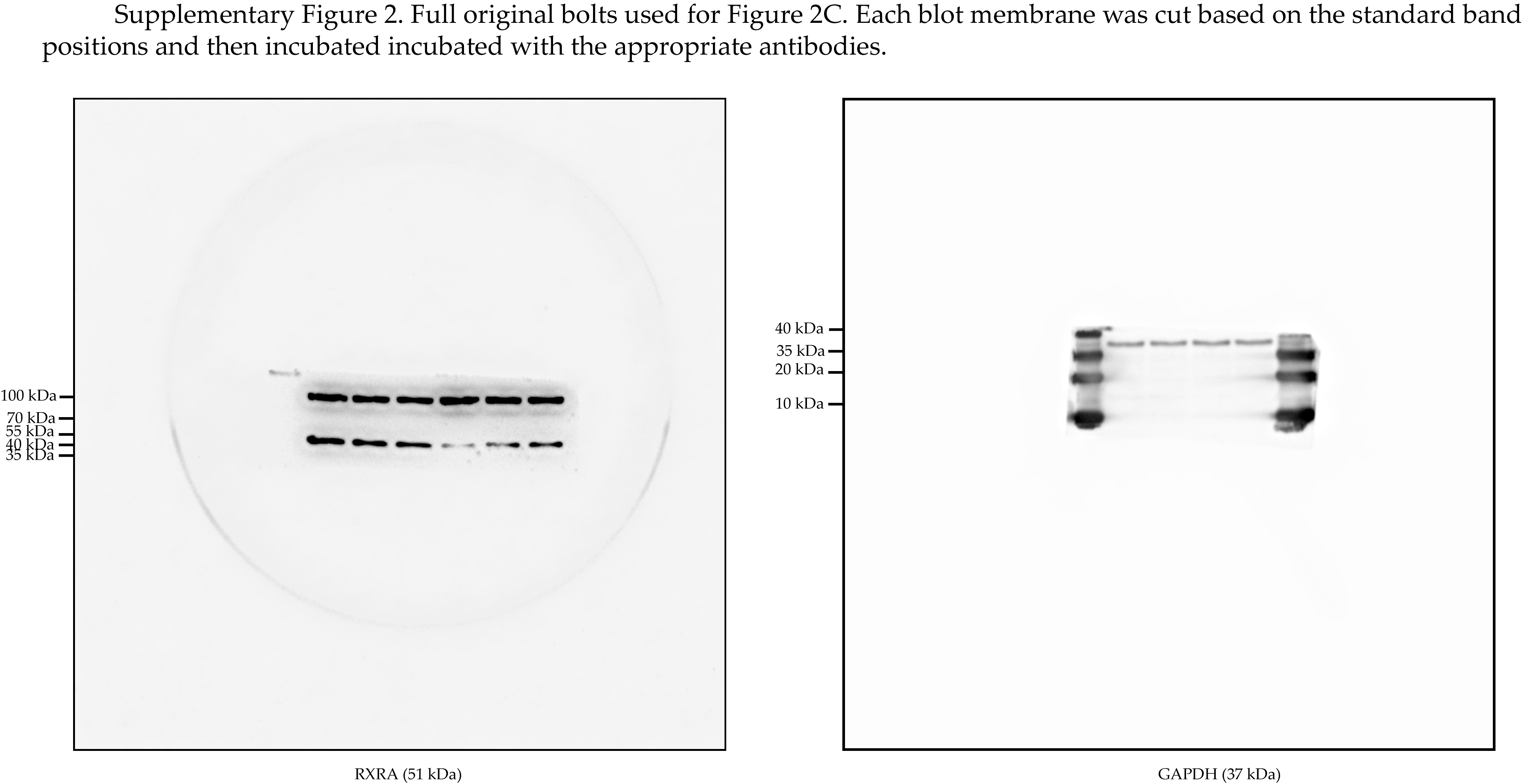

Supplement: Supplementary file 1 [file animals-13-00680-s001.zip › Supplementary materials/Figure S2-Full original bolts used for Figure 2C.tif]
